# Fe_3_O_4_ hard templating to assemble highly wrinkled graphene sheets into hierarchical porous film for compact capacitive energy storage†

**DOI:** 10.1039/c9ra02132a

**Published:** 2019-06-27

**Authors:** Hua Fang, Fanteng Meng, Ji Yan, Gao-yun Chen, Linsen Zhang, Shide Wu, Shichao Zhang, Lizhen Wang, Yongxia Zhang

**Affiliations:** School of Material and Chemical Engineering, Zhengzhou University of Light Industry Zhengzhou 450001 PR China fh@zzuli.edu.cn jiyan@zzuli.edu.cn; Institute of Chemical Defense Beijing 102205 PR China; School of Materials Science and Engineering, Beihang University Beijing 100191 PR China csc@buaa.edu.cn

## Abstract

Highly wrinkled graphene film (HWGF) with high packing density was synthesized by combining an electrostatically self-assembling process, a vacuum filtration-induced film assembling process and capillary compression. Fe_3_O_4_ nanoparticles were used as a low-cost and environment-friendly hard template. Hierarchical porosity and high packing density were achieved with the aid of capillary compression in the presence of Fe_3_O_4_ nanoparticles. This strategy enables integration of highly wrinkled graphene sheets to form highly compact carbon electrodes with a continuous ion transport network. The generated HWGF exhibited a high packing density of 1.53 g cm^−3^, a high specific surface area of 383 m^2^ g^−1^ and a hierarchically porous structure. The HWGF delivered a high capacitance of 242 F g^−1^ and 370 F cm^−3^ at 0.2 A g^−1^ in 6 M KOH aqueous electrolyte system with excellent rate capability (202 F g^−1^ and 309 F cm^−3^ retained at 20 A g^−1^). The capacity retention rate reached 97% after 10 000 cycles at 1 A g^−1^. The HWGF-based supercapacitor exhibited a high energy density of 17 W h kg^−1^ at the power density of 49 W kg^−1^. Such high capacitive performances could be attributed to the highly dense but porous graphene assemblies composed of highly wrinkled graphene sheets.

## Abstract 2

A facile strategy was reported to synthesize highly wrinkled graphene film, exhibiting high packing density and excellent capacitive performance.

## Graphical abstract

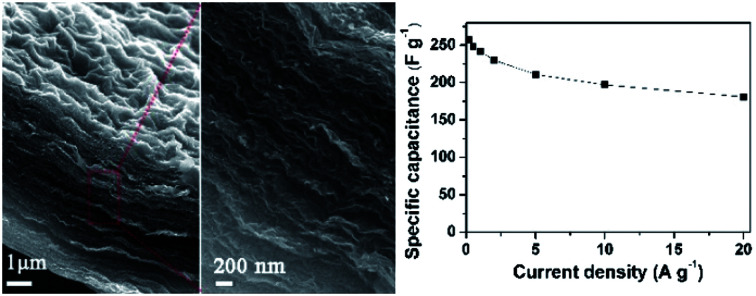

## Introduction

Electrochemical capacitors (ECs), also known as supercapacitors, store energy by charging electrical double layers through highly reversible ion adsorption on the surface of high-surface-area electrodes, generally made from porous carbon. Due to their fast charging capability and long life span, supercapacitors are attractive in powering mobile electronics, electric vehicles (EVs) and storing renewable energy for power grids. Graphene is recognized as a promising electrode material for high-performance supercapacitors, due to its flexibility, excellent electrical conductivity, and high theoretical specific surface area (∼2630 m^2^ g^−1^).^1,2^ However, graphene sheets are inclined to re-aggregate and stack together because of high surface area and strong van der Waals forces. This aggregation decreases their specific surface area and restricts the accessibility of electrolyte ions to their surface, thus leading to a deteriorated performance.^3,4^

To overcome such limitation, researches have focused on three-dimensional (3D) porous graphene architecture, such as highly-crumpled graphene,^5–9^ graphene foam,^10–12^ graphene gel,^13–16^ thermal exfoliated graphene,^17,18^ and chemical activated graphene.^19–21^ Typically, most of those graphene based materials were composed of interlinked graphene nanosheets and delivered high specific surface area, excellent conductivity and open ion channels. Unfortunately, the above mentioned graphene assemblies usually have a low packing density, ranging from 0.05 to 0.75 g cm^−3^.^22^ Such low packing density resulted in small volumetric capacitance, which has become one of the major limitations for novel nanocarbons finding real applications in commercial electrochemical energy storage devices.^23^ Y. Gogotsi and P. Simon have profoundly explored the small volumetric capacitances for the nanomaterials.^24^ As a result, the volumetric energy density was recently recommended to be a more reliable parameter than the gravimetric one to evaluate the real potential of a porous carbon for high-performance supercapacitors.^24–26^ However, highly porous nature, which is crucial for high ion-accessible surface area and low ion transport resistance of carbon electrode, seems to be irreconcilable with high packing density. Porous yet densely packed graphene electrodes, which are required to realize high-density electrochemical capacitive energy storage, have proved to be very challenging to produce.

Recently, many researches have been focused on highly dense but porous graphene assemblies, opening up new ways to address this challenge. Yang *et al.* reported a graphene hydrogel films, with a metastable and adaptive pore structure, can be compressed irreversibly by capillary pressure to increase the packing density through controlled removal of volatile solvent trapped in the gel.^22^ Volumetric capacitances reached 255.5 F cm^−3^ in aqueous electrolyte and 261.3 F cm^−3^ in organic electrolyte at 0.1 A g^−1^, which were much higher than those of the existing porous carbon materials. The packing density can be increased up to ∼1.33 g cm^−3^, nearly double that of the traditional activated porous carbon (0.5 to 0.7 g cm^−3^).^27^ Tao *et al.* reported a highly dense but porous graphene-based monolithic carbon with a very high density of 1.58 g cm^−3^, which is 70% of the theoretical density of graphite (2.2 g cm^−3^).^23^ Such graphene assembly was produced by an evaporation-induced drying of a graphene hydrogel and constructed of compactly interlinked nanosheets, exhibiting a volumetric capacitance up to 376 F cm^−3^ in aqueous electrolyte.

In this article, we report a low-cost iron oxide hard template strategy to create highly wrinkled graphene film (HWGF) with hierarchical pore structure and high packing density, balancing these two opposing characteristics. Such HWGF can be readily formed by vacuum filtration process and the following heat treatment process with the aid of capillary compression in the presence of Fe_3_O_4_ nanoparticles. The generated porous yet densely packed HWGF was binder-free and showed robust chemical and mechanical stability. The hierarchical structure facilitates fast ion transport and thus enhanced electrochemical performances were achieved, such as capacitance, cycling stability and rate capability. The high packing density resulted in high volumetric capacitance, facilitating real applications in commercial electrochemical energy storage devices. It should be mentioned that the fabrication process is facile, low-cost and scalable, opening up a new way for the rational design and effective synthesis of highly dense but porous graphene assemblies for high-performance supercapacitors.

## Experimental

### Fabrication of CCNF

All the chemicals were used in analytical grades without purification. Graphite oxide was synthesized from natural graphite powder by the modified Hummers' method.^28^

The prepared graphite oxide was ultrasonically dispersed in deionized water for 1 h to obtain graphene oxide (GO) hydrosol (0.1 g L^−1^).

For preparing Fe(OH)_3_ colloid solution, 23 mL of urea solution (0.2 M) was added into 100 mL of FeCl_3_ solution (0.0155 M). Then, the mixed solution was heated up to 80 °C in a water bath and kept at 80 °C for 40 min under magnetic stirring. The obtained Fe(OH)_3_ colloid solution was cooled to room temperature for further experiment.

The HWGF was prepared by the following processes. First, the prepared GO hydrosol was added dropwise into the prepared Fe(OH)_3_ colloid solution under magnetic stirring. The volume ratio of GO hydrosol and Fe(OH)_3_ colloid solution was set as 1 : 2. A flocculent precipitate was formed within a few minutes and a Fe(OH)_3_@GO hybrid film was obtained by vacuum filtration through a microfiltration devices. Secondly, the Fe(OH)_3_@GO hybrid film were subjected to heat treatment at 300 °C in a tube-furnace under N_2_ atmosphere, leading to the formation of Fe_3_O_4_@rGO hybrid film. Finally, the Fe_3_O_4_@rGO hybrid film was washed by diluted hydrochloric acid to remove Fe_3_O_4_ and the HWGF was obtained.

### Materials characterization

Surface morphologies were characterized by using a JEOL JSM-7001F scanning electron microscope (SEM). Phase was examined by using an X-ray diffractometer (XRD, Bruker Axs DS Advance) with Cu Kα radiation and a Raman spectroscopy (LabRam HR HORIBA) with 514 nm wavelength laser for detection. Nitrogen adsorption/desorption test was performed at 77 K on a specific surface and porosity analyzer (BELSORP-Mini II). Specific surface area and pore size distribution were calculated by the conventional Brunauer–Emmett–Teller (BET) and Barrett–Joyner–Halenda (BJH) method, respectively.

### Electrochemical measurements

The electrochemical tests were performed in two-electrode supercapacitors, in which a separator, soaked in a 6 M KOH aqueous solution, was sandwiched between two HWGF electrodes. The HWGF electrodes were prepared by pressing the HWGF film onto a nickel foam disk at 20 MPa (1 cm × 1 cm as a current collector, with a nickel tape for connection) and then drying 6 h at 80 °C under vacuum. Cyclic voltammetry (CV) measurements were performed between 0–1 V at different scan rates from 5 to 100 mV s^−1^ on a CHI 604 electrochemical workstation (Shanghai, China). Galvanostatic charge/discharge (GCD) tests were performed between 0.01–1 V at different current densities from 0.2 to 20.0 A g^−1^ on a Neware 2001 battery test system (Neware Instruments). Electrochemical impedance spectroscopy (EIS) tests were performed at open-circuit potential with amplitude of 5 mV from 100 kHz to 10 mHz.

The specific capacitance, energy density and power density of the HWGF electrode was calculated according to the following formula:^29^*C*_s_ = 4*C* = 2*I*Δ*t*/(Δ*Vm*)*E* = 1/2*C*Δ*V*^2^*P* = *E*/Δ*t*where *C*_s_ is the capacitance of single electrode (F g^−1^), *C* is the capacitance of double electrode (F g^−1^), *I* is the discharge current (A), Δ*t* is the discharge time (s), Δ*V* is the potential change in discharge (V), *m* is the mass of the carbon material on single electrodes (g), *E* is energy density and *P* is power density.

## Results and discussion

**Scheme 1:**
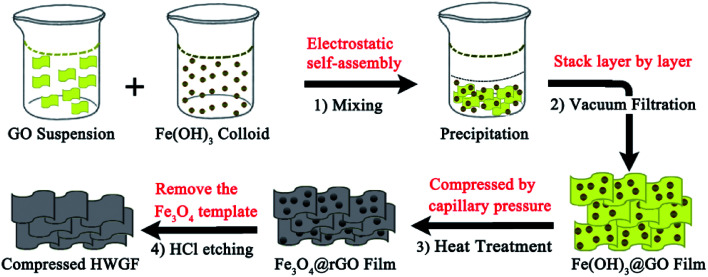
The schematics of the fabrication strategy of HWGF.

As schematically illustrated in Scheme 1, the hierarchical HWGF with high packing density was fabricated by using low-cost iron oxide as hard template method. Firstly, the negatively charged GO colloid was dropped into the positively charged Fe(OH)_3_ colloid, during which process the Fe(OH)_3_ colloid particles were electrostatically self-assembled onto the surface of GO sheets. As a result, a flocculent precipitate was produced. As shown in Fig. S1,† the flocculent precipitate can be formed with the different volume ratio of GO hydrosol and Fe(OH)_3_ colloid solution ranging from 1 : 1 to 1 : 20. In this paper, the volume ratio of GO hydrosol and Fe(OH)_3_ colloid solution was set as 1 : 2.

Secondly, the produced flocculent precipitate substance is self-assembled into Fe(OH)_3_@GO hybrid film in the following vacuum filtration-induced assembly process. Thirdly, the Fe(OH)_3_@GO hybrid film is converted to Fe_3_O_4_@rGO film *via* the heat treatment process. Finally, the HWGF, as shown in Fig. S2† is achieved *via* the acid washing process. Such HWGF can be highly compressed during the heat treatment process with the aid of capillary compression. Thanks to the presence of Fe_3_O_4_ nanoparticles, the HWGF could retain its porous structure in some extent, endowing graphene sheets with highly wrinkled morphology.

**Fig. 1:**
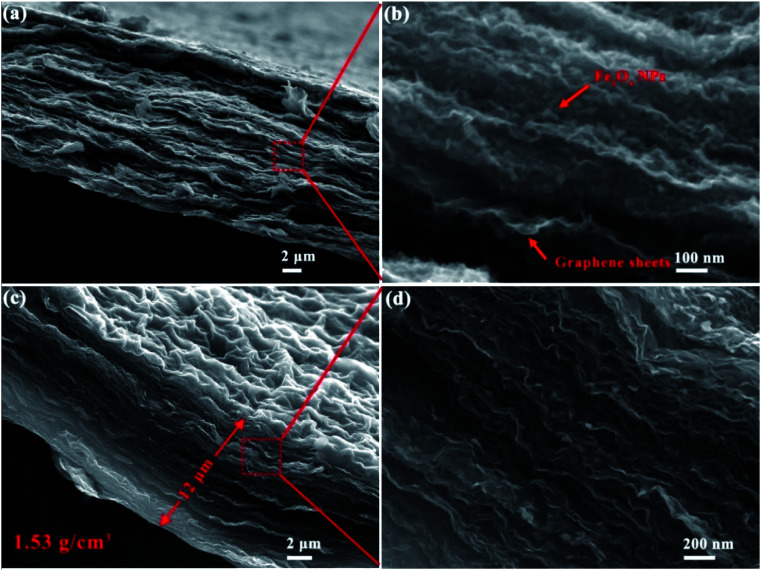
Section view SEM images of the Fe_3_O_4_@rGO hybrid film (a and b) and the HWGF (c and d).

As shown by Fig. 1a and b, the graphene sheets in the films stacked in a nearly face-to-face fashion and Fe_3_O_4_ nanoparticles (NPs) are homogeneously embedded between these nanosheets. Fe_3_O_4_ nanoparticles acted as a low cost hard template and show steric hindrance effect, endowing graphene sheets with highly wrinkled morphology. As shown in Fig. 1c and d, HWGF was generated after removal of the Fe_3_O_4_ nanoparticles. The HWGF show highly wrinkled yet densely packed morphology, resulted from the joint action of steric hindrance effect of Fe_3_O_4_ nanoparticles and the capillary compression during the heat treatment process. As a result, the flexible HWGF exhibited a comparatively high packing density of 1.53 g cm^−3^, which is calculated based on its areal density (1.84 mg cm^−2^) and average film thickness (12 μm).

It has been reported that capillary compression rather than thermal annealing is responsible for the crumpled graphene sheets. The crumpled graphene is also stabilized by plastically deformed ridges, and thus does not unfold or collapse during various types of solution processing or chemical or heating treatments.^30^ As shown in Fig. 1c and d of this research, the graphene sheets show a highly crumpled morphology, resulting in plenty of deformed ridges. The deformed ridges is believed to facilitate the stability of the graphene film (HWGF), preventing the HWGF film from cracking.

It should be mentioned that the Fe_2_O_3_ NPs shown in side view (Fig. 1b) are small, while the Fe_2_O_3_ NPs shown in top view (Fig. S3†) exhibit a larger size (>20 nm). Such obvious morphological differences may be caused by steric hindrance effect of graphene. For the Fe_2_O_3_ NPs anchored on the surface of the highly wrinkled graphene film, the phenomenon of aggregation and crystal growth should occur during the heat treatment process. However, for the Fe_2_O_3_ NPs enveloped in the highly wrinkled graphene sheets, the phenomenon of aggregation and crystal growth should not occur during the heat treatment process due to the steric hindrance effect of graphene sheet.

**Fig. 2:**
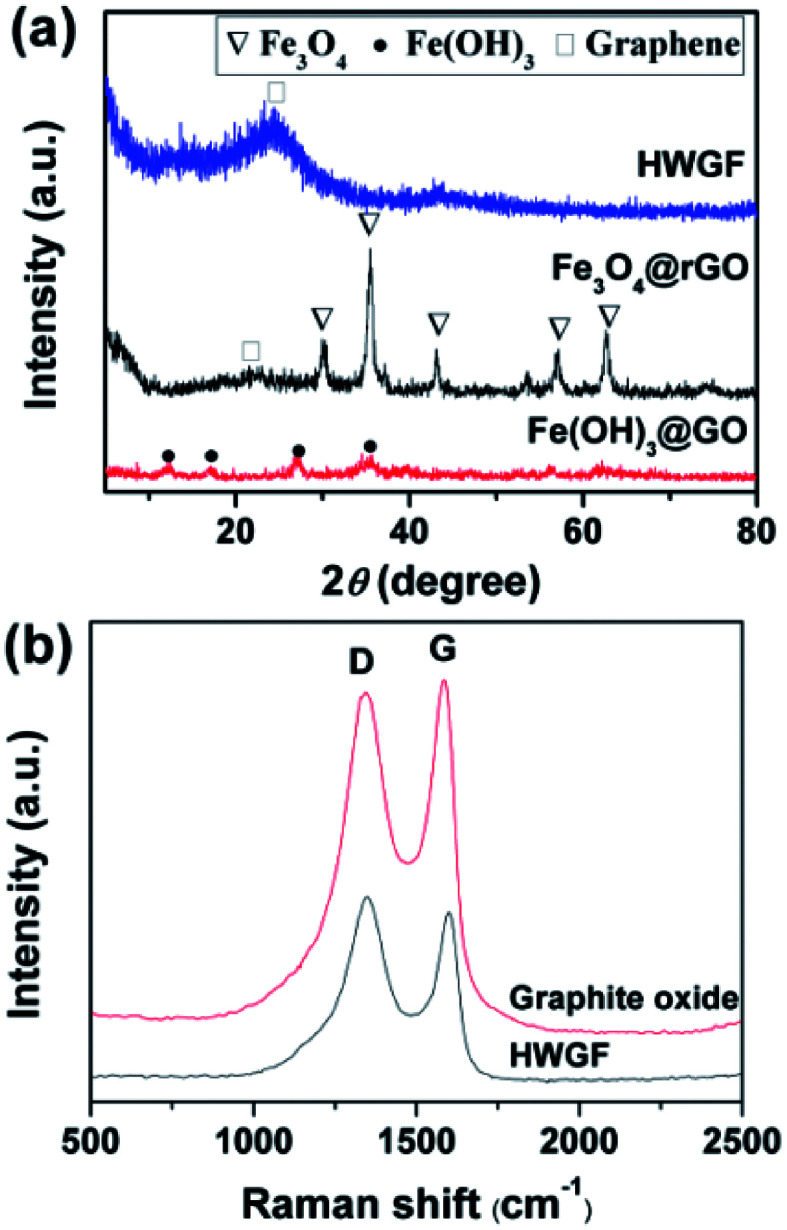
(a) XRD patterns of the Fe(OH)_3_@GO hybrid film, Fe_3_O_4_@rGO hybrid film and HWGF, and (b) Raman spectra of the HWGF and the graphite oxide.

XRD tests were performed to investigate the phase of Fe(OH)_3_@GO hybrid film, Fe_3_O_4_@rGO hybrid film and HWGF. As shown in Fig. 2a, the Fe(OH)_3_@GO hybrid film shows XRD peaks at 12.4°, 17.2°, 27.0°, and 35.7°, which can be well indexed to the (110), (200), (310) and (211) planes of Fe(OH)_3_ (JCPDS card no. 34-1266). The Fe_3_O_4_@rGO hybrid film shows XRD peaks of 30.2, 35.4, 43.2, 57.3 and 62.9°, which are well agreed with (220), (311), (400), (511) and (440) crystal planes of face-centered cubic Fe_3_O_4_ (JCPDS standard card no. 86-1354).^31^ The HWGF shows a broad peak centered at 23.9°, which is the characteristic diffraction peak of graphene sheets. The above mentioned results were also supported by Raman measurements.

As shown in Fig. 2b, the Raman spectra of HWGF and graphite oxide exhibit D band at around 1340 cm^−1^ and G band at about 1600 cm^−1^. The D band corresponds to the structural defects of graphitic domains, whereas the G band is associated with the ordered sp^2^ bonded carbon.^10^ The peak intensity ratio of D to G (*I*_D_/*I*_G_) of the graphite oxide (0.974) is consistent with the reported rations for graphite oxide materials.^17^ The *I*_D_/*I*_G_ value of the HWGF (1.075) is closed to those of the reported amorphous carbon materials, indicating that the HWGF is mainly composed of amorphous carbon.^32^ The ratio of D and G band intensities (*I*_D_/*I*_G_) in HWGF (*I*_D_/*I*_G_ = 1.075) is slightly higher than that in graphite oxide sample (*I*_D_/*I*_G_ = 0.974), which was attributed to the defects created by the removal of oxygen moieties of graphite oxide.^33^

**Fig. 3:**
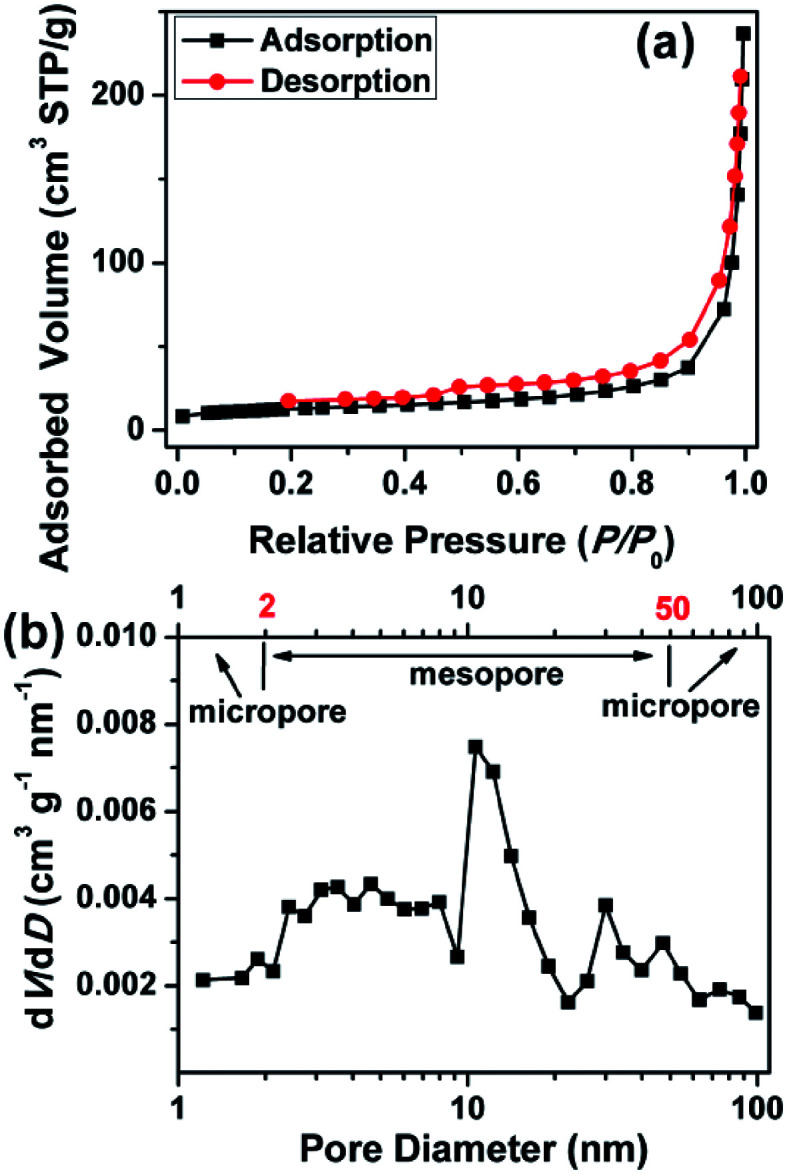
(a) N_2_ adsorption isotherms and (b) pore size distributions calculated by BJH method of the HWGF.

As shown in Fig. 3a, the HWGF shows a combined I/IV type adsorption–desorption isotherms with a H3 hysteresis loop, indicating the presence of slit-like pores.^34^ The HWGF show a high specific surface area of 383 m^2^ g^−1^, an average pore size of 27.7 nm and a total pore volume of 0.2652 m^3^ g^−1^. As shown in Fig. 3b, the HWGF show wide pore size distribution including micropore (<2 nm), mesopore (2–50 nm) and macropore (>50 nm). Hierarchical porosity is known to provide large accessible surface area and short ion pathways and thus improved electrochemical performances for carbon electrode materials for supercapacitors.^35^

**Fig. 4:**
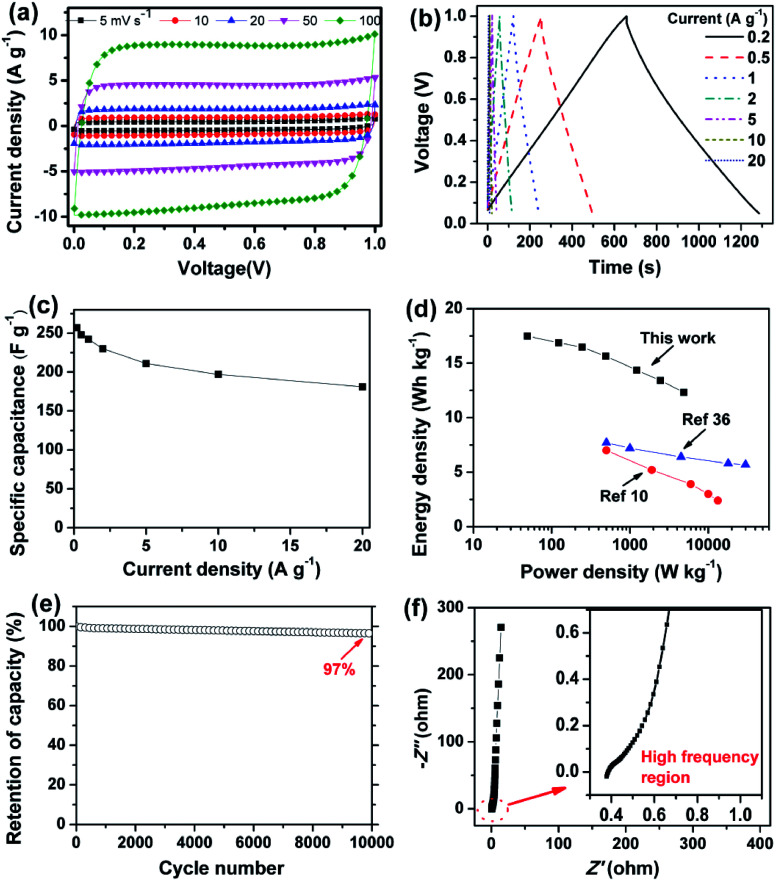
Electrochemical capacitive performances of the HWGF based two-electrode supercapacitors in 6 M KOH. (a) CV profiles at scan rates from 5 to 100 mV s^−1^. (b) GCD curves at different current densities from 0.2 to 20 A g^−1^. (c) Rate performances. (d) Ragone plot of the two-electrode supercapacitor (inset shows the Ragone plots of the previously reported graphene based symmetric supercapacitors^10,36^). (e) Cycling stability at 1 A g^−1^. (f) Nyquist plots (inset shows the high frequency region).

As designed, the HWGF proved to be an ideal electrode material for supercapacitors. As shown in Fig. 4a, the HWGF electrode retains a nearly rectangular shaped CV curves at scan rates from 5 to 100 mV s^−1^, indicating that the capacitance mainly comes from electric double layer capacitance (EDLC). As shown in Fig. 4b, the HWGF electrode exhibits symmetric triangle shaped GCD curves under different current densities from 0.2 to 20 A g^−1^, indicating its small resistance and excellent rate capability.

The specific capacitance is calculated from the GCD test results. As shown in Fig. 4c, the HWGF electrode showed a high capacitance of 257 F g^−1^ at 0.2 A g^−1^ and 181 F g^−1^ at 20 A g^−1^. Based on its high packing density of 1.53 g cm^−3^, the volumetric capacitances of the HWGF can reach as high as 370 F cm^−3^ at 0.2 A g^−1^ and 309 F cm^−3^ at 20 A g^−1^. The capacitance retention rate reaches as high as 70% when the current density increased by 100 times from 0.2 to 20 A g^−1^, indicating the superior rate capability of the HWGF.

Based on the rate performance shown in Fig. 4c, the energy densities and power densities were calculated for the two-electrode symmetric supercapacitor. As shown in Fig. 4d, the symmetric supercapacitor based on HWGF delivers a high energy density of 17.5 W h kg^−1^ at a power density of 49 W kg^−1^ and 12.3 W h kg^−1^ at 4900 W kg^−1^. These data are highly comparable to the previously reported graphene based symmetric supercapacitors, such as 3D porous graphene (7.0 W h kg^−1^ at 500 W kg^−1^)^10^ and graphene hydrogel (5.7 W h kg^−1^ at 30 kW kg^−1^).^36^

GCD cycle test was performed at 1 A g^−1^ to investigate the cycle stability, which is critical for its practical application in supercapacitors. As depicted in Fig. 4e, the HWGF electrode show a capacitance retention rate of 97% after 10 000 GCD cycles, proving its superior electrochemical stability and reversibility. Furthermore, as shown in Fig. 4f, the HWGF electrode shows an almost vertical line at the low frequency region, demonstrating its fast ion diffusion and ideal EDLC behavior.^17^

## Conclusions

In summary, highly wrinkled graphene film (HWGF) was synthesized by using Fe_3_O_4_ nanoparticles as low-cost and environment friendly hard template. The HWGF show highly wrinkled yet densely packed morphology, resulted from the joint action of steric hindrance effect of Fe_3_O_4_ nanoparticles and the capillary compression during the heat treatment process. The generated HWGF exhibited a high packing density of 1.53 g cm^−3^, a high specific surface area of 383 m^2^ g^−1^ and a hierarchically porous structure. The high packing density resulted in high volumetric capacitance, facilitating real applications in commercial electrochemical energy storage devices. The HWGF delivered a high capacitance of 242 F g^−1^ and 370 F cm^−3^ at 0.2 A g^−1^ in 6 M KOH aqueous electrolyte system, with excellent rate capability (202 F g^−1^ and 309 F cm^−3^ retained at 20 A g^−1^) and cycle stability (97% of its initial capacitance retained after 10 000 cycles at 1 A g^−1^). In brief, the fabrication process is facile, low-cost and scalable, opening up a promising way for the rational design and effective synthesis of highly dense but porous graphene assemblies for compact capacitive energy storage.

## Conflicts of interest

There are no conflicts to declare.

## Supplementary Material

RA-009-C9RA02132A-s001

## References

[cit1] Zhang Q., Wang Y., Zhang B., Zhao K., He P., Huang B. (2018). Carbon.

[cit2] He X., Li X., Ma H., Han J., Zhang H., Yu C., Xiao N., Qiu J. (2017). J. Power Sources.

[cit3] Li X. J., Xing W., Zhou J., Wang G. Q., Zhuo S. P., Yan Z. F., Xue Q. Z., Qiao S. Z. (2014). Chem.–Eur. J..

[cit4] You B., Wang L., Li N., Zheng C. (2014). ChemElectroChem.

[cit5] Yang X., Cheng C., Wang Y., Qiu L., Li D. (2013). Science.

[cit6] Yan J., Wang Q., Wei T., Jiang L., Zhang M., Jing X., Fan Z. (2014). ACS Nano.

[cit7] Yoon Y., Lee K., Baik C., Yoo H., Min M., Park Y., Lee S. M., Lee H. (2013). Adv. Mater..

[cit8] Wen Z., Wang X., Mao S., Bo Z., Kim H., Cui S., Lu G., Feng X., Chen J. (2012). Adv. Mater..

[cit9] Xiong Z., Liao C., Wang X. (2014). J. Mater. Chem. A.

[cit10] Li T., Li N., Liu J., Cai K., Foda M. F., Lei X., Han H. (2015). Nanoscale.

[cit11] Chen C.-M., Zhang Q., Huang C.-H., Zhao X.-C., Zhang B.-S., Kong Q.-Q., Wang M.-Z., Yang Y.-G., Cai R., Sheng Su D. (2012). Chem. Commun..

[cit12] Chen Z., Ren W., Gao L., Liu B., Pei S., Cheng H. M. (2011). Nat. Mater..

[cit13] Shao Q., Tang J., Lin Y., Li J., Qin F., Yuan J., Qin L.-C. (2015). J. Power Sources.

[cit14] Qian Y., Ismail I. M., Stein A. (2014). Carbon.

[cit15] Xu Y., Lin Z., Huang X., Wang Y., Huang Y., Duan X. (2013). Adv. Mater..

[cit16] Chen J., Sheng K., Luo P., Li C., Shi G. (2012). Adv. Mater..

[cit17] Tian W., Gao Q., Tan Y., Zhang Y., Xu J., Li Z., Yang K., Zhu L., Liu Z. (2015). Carbon.

[cit18] El-Kady M. F., Strong V., Dubin S., Kaner R. B. (2012). Science.

[cit19] Wang P., He H., Xu X., Jin Y. (2014). ACS Appl. Mater. Interfaces.

[cit20] Zhu Y., Murali S., Stoller M. D., Ganesh K. J., Cai W., Ferreira P. J., Pirkle A., Wallace R. M., Cychosz K. A., Thommes M., Su D., Stach E. A., Ruoff R. S. (2011). Science.

[cit21] Zhang L. L., Zhao X., Stoller M. D., Zhu Y., Ji H., Murali S., Wu Y., Perales S., Clevenger B., Ruoff R. S. (2012). Nano Lett..

[cit22] Yang X., Cheng C., Wang Y., Qiu L., Li D. (2013). Science.

[cit23] Tao Y., Xie X., Lv W., Tang D.-M., Kong D., Huang Z., Nishihara H., Ishii T., Li B., Golberg D., Kang F., Kyotani T., Yang Q.-H. (2013). Sci. Rep..

[cit24] Gogotsi Y., Simon P. (2011). Science.

[cit25] Murali S., Quarles N., Zhang L. L., Potts J. R., Tan Z., Lu Y., Zhu Y., Ruoff R. S. (2013). Nano Energy.

[cit26] Simon P., Gogotsi Y. (2013). Acc. Chem. Res..

[cit27] Burke A. (2007). Electrochim. Acta.

[cit28] Hummers W. S., Offeman R. E. (1958). J. Am. Chem. Soc..

[cit29] Zhang X., Wang J., Yu Z., Wang R., Xie H. (2009). Mater. Lett..

[cit30] Luo J., Jang H. D., Sun T., Xiao L., He Z., Katsoulidis A. P., Kanatzidis M. G., Gibson J. M., Huang J. (2011). ACS Nano.

[cit31] Liu Y., Zhan Y., Ying Y., Peng X. (2016). New J. Chem..

[cit32] Zhao K. M., Liu S. Q., Ye G. Y., Gan Q. M., Zhou Z., He Z. (2018). J. Mater. Chem. A.

[cit33] Sahoo S., Shim J.-J. (2017). ACS Sustainable Chem. Eng..

[cit34] Wang Q., Yan J., Wang Y., Wei T., Zhang M., Jing X., Fan Z. (2014). Carbon.

[cit35] Zhu C., Liu T., Qian F., Han T. Y.-J., Duoss E. B., Kuntz J. D., Spadaccini C. M., Worsley M. A., Li Y. (2016). Nano Lett..

[cit36] Zhang L., Shi G. (2011). J. Phys. Chem. C.

