# Supplementary material for: Fe3O4 hard templating to assemble highly wrinkled graphene sheets into hierarchical porous film for compact capacitive energy storage
Source: RSC Adv. 2019 Jun 27;9(35):20107–12. doi: 10.1039/c9ra02132a (PMC9065504; doi:10.1039/c9ra02132a)
Supplement: RA-009-C9RA02132A-s001 [file RA-009-C9RA02132A-s001.pdf]

Electronic Supplementary Material (ESI) for RSC Advances.
This journal is © The Royal Society of Chemistry 2019

Supplementary Information

Fe_3O_4 Hard Templating to Assemble Highly Wrinkled Graphene Sheets into Hierarchical Porous Film for Compact Capacitive Energy Storage

Hua Fang*,[a], Fanteng Meng[a], Ji Yan*,[a], Gao-yun Chen[b], Linsen Zhang[a], Shide Wu[a], Shichao Zhang*,[c], Lizhen Wang[a], Yongxia Zhang[a]

[a] School of Material and Chemical Engineering, Zhengzhou University of Light Industry, Zhengzhou 450001, PR China

b. Institute of Chemical Defense, Beijing, 102205, PR China

[c] School of Materials Science and Engineering, Beihang University, Beijing, 100191, PR China

Figure S1

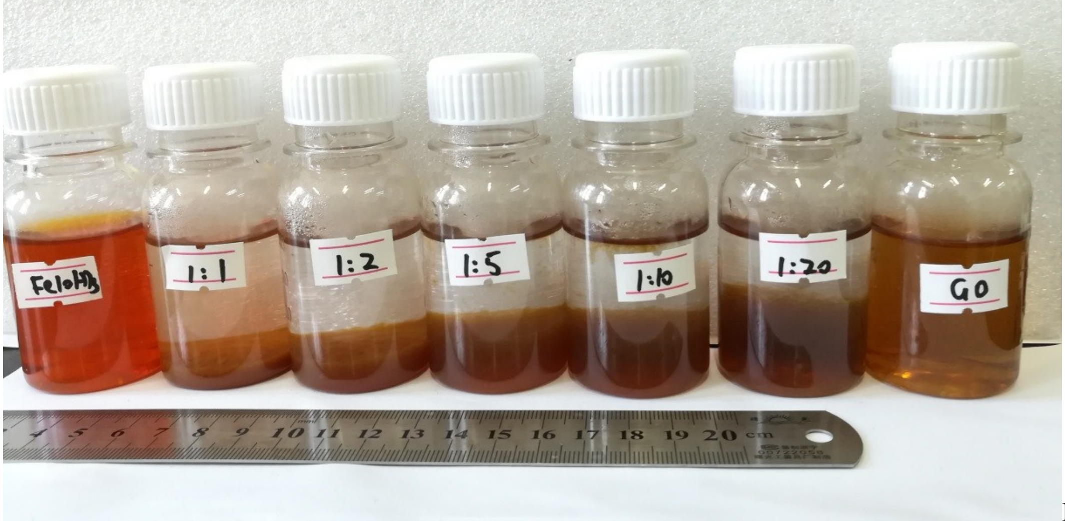

Fig. S1 The flocculent precipitate formed with the different volume ratio of GO hydrosol and $Fe(OH)_3$ colloid solution ranging from 1:1 to 1:20 (the photo was taken after the hybrids were subjected to 9 hours standing).

* Corresponding author. E-mail addresses: fh@zzuli.edu.cn (Hua Fang), jiyan@zzuli.edu.cn (Ji Yan), csc@buaa.edu.cn (Shichao Zhang)

Figure S2

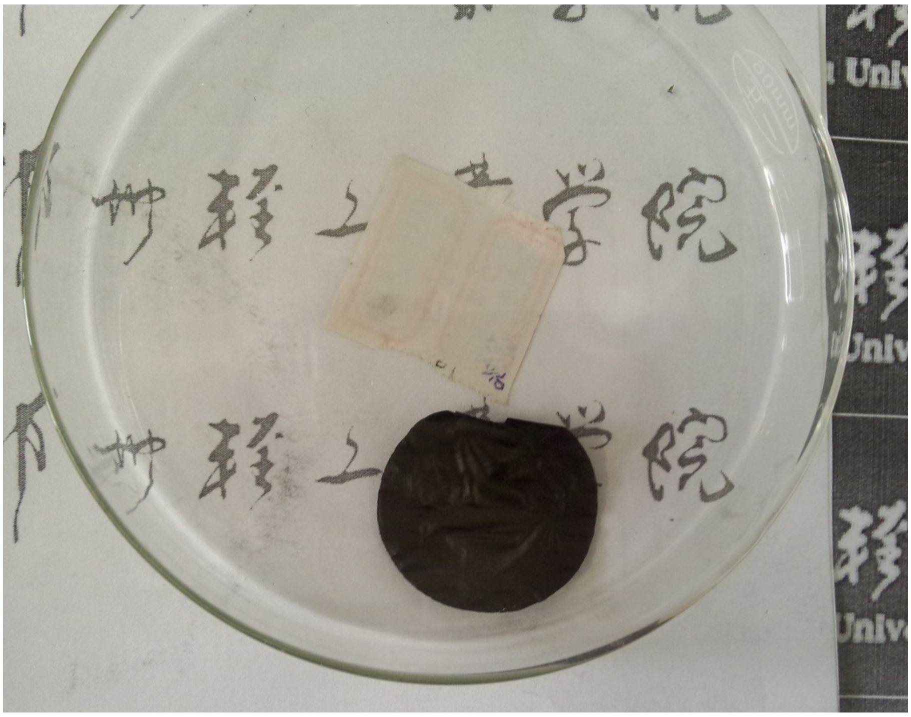

Fig. S2 The image of the generated highly wrinkled graphene film (HWGF)

Figure S3

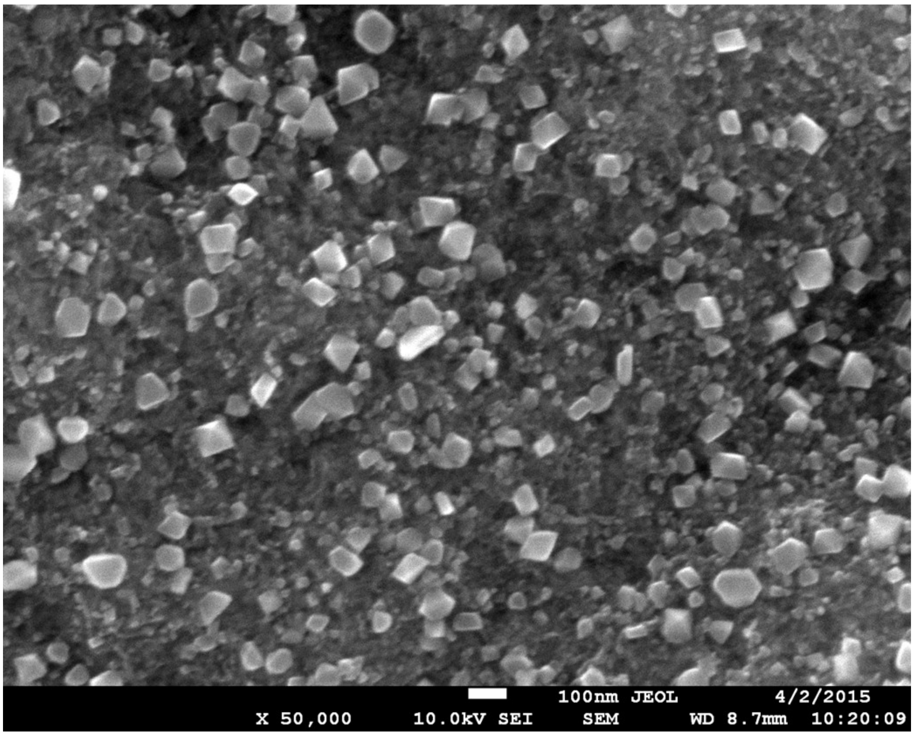

Fig. S3 Top view SEM image of the Fe_3O_4@rGO hybrid film
